# Aggression Results in the Phosphorylation of ERK1/2 in the Nucleus Accumbens and the Dephosphorylation of mTOR in the Medial Prefrontal Cortex in Female Syrian Hamsters

**DOI:** 10.3390/ijms24021379

**Published:** 2023-01-10

**Authors:** Johnathan M. Borland, Desarae A. Dempsey, Anna C. Peyla, Megan A. L. Hall, Abigail L. Kohut-Jackson, Paul G. Mermelstein, Robert L. Meisel

**Affiliations:** 1 Department of Neuroscience, University of Minnesota, Minneapolis, MN 55455, USA; 2 Stark Neurosciences Research Institute, Indiana University School of Medicine, Indianapolis, IN 46202, USA

**Keywords:** social interaction, synaptic plasticity, glutamate, fragile X mental retardation protein, dominance, prefrontal cortex, caudate putamen, reward

## Abstract

Like many social behaviors, aggression can be rewarding, leading to behavioral plasticity. One outcome of reward-induced aggression is the long-term increase in the speed in which future aggression-based encounters is initiated. This form of aggression impacts dendritic structure and excitatory synaptic neurotransmission in the nucleus accumbens, a brain region well known to regulate motivated behaviors. Yet, little is known about the intracellular signaling mechanisms that drive these structural/functional changes and long-term changes in aggressive behavior. This study set out to further elucidate the intracellular signaling mechanisms regulating the plasticity in neurophysiology and behavior that underlie the rewarding consequences of aggressive interactions. Female Syrian hamsters experienced zero, two or five aggressive interactions and the phosphorylation of proteins in reward-associated regions was analyzed. We report that aggressive interactions result in a transient increase in the phosphorylation of extracellular-signal related kinase 1/2 (ERK1/2) in the nucleus accumbens. We also report that aggressive interactions result in a transient decrease in the phosphorylation of mammalian target of rapamycin (mTOR) in the medial prefrontal cortex, a major input structure to the nucleus accumbens. Thus, this study identifies ERK1/2 and mTOR as potential signaling pathways for regulating the long-term rewarding consequences of aggressive interactions. Furthermore, the recruitment profile of the ERK1/2 and the mTOR pathways are distinct in different brain regions.

## 1. Introduction

Society has historically viewed aggressive behavior as maladaptive. However, aggression is a natural behavior commonly displayed throughout the animal kingdom and is essential for the allocation of resources, fitness and mental health [1,2,3,4]. Furthermore, in addition to the adaptive consequences of aggression being understudied, aggression is also historically understudied in females [5]. In fact, due to male-centered societal constructs and the poor to non-existent models for eliciting aggression in female mice and rats, almost nothing is known about the neural mechanisms of aggression in females [6]. 

As in other species and across both sexes, aggressive interactions are rewarding in female Syrian hamsters based on observations that females will display an increase in the amount of time spent in environments associated with aggressive experiences and will seek out social stimuli to engage in aggressive interactions [7,8]. These rewarding experiences become part of a feed-forward process through which there is an increase in the desire and motivation to reinitiate aggression in future encounters. Female Syrian hamsters with aggressive experience are quicker to initiate attacks compared to females that do not have a history of aggressive experience [9,10]. 

Aggressive interactions in female hamsters result in neuroplastic changes within the nucleus accumbens (NAc). These include long-term increases in mushroom-like dendritic spines on medium spiny neurons (MSNs) and an increase in postsynaptic density protein 95 (PSD-95), metabotropic glutamate receptor 5 (mGluR5), and α-amino-3-hydroxy-5-methyl-4-isoxazolepropionic acid receptor (AMPAR) protein [9,11]. The activation of mGluR5 is required for both the increase in the expression of PSD-95 and the decrease in the latency to display aggression resulting from recurring aggressive interactions [10]. However, almost nothing is known about the intracellular signaling events that underlie these persistent synaptic adaptations and behavioral plasticity. 

**Figure 1:**
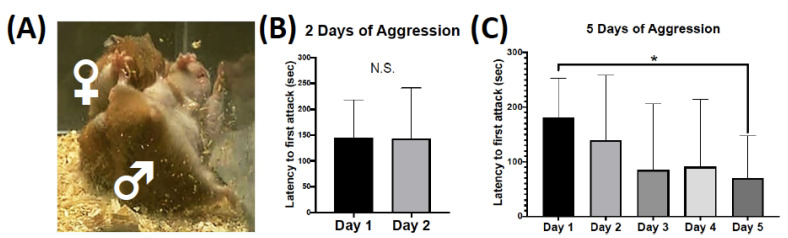
Aggressive behavior over two and five consecutive days of aggressive interactions (5 min each). (**A**) Image of an adult female Syrian hamster biting the lower flank of an intruding adult male Syrian hamster. (**B**) There was no change in the latency to initiate an attack for female Syrian hamsters that experienced two consecutive days of aggressive interactions (non-significant = N.S.) (Days 1–2, n = 11). (**C**) There was a decrease in the latency to initiate an attack for female Syrian hamsters that experienced five consecutive days of aggressive interactions (* *p* < 0.05). (Days 1–5, n = 9). Data are presented as mean +/− standard deviation.

These dramatic changes in synaptic structure and excitatory neurotransmission are most likely due to short-term changes in cell transcription and translation [12,13,14]. Interestingly, it has been shown that the translational events for synaptic plasticity can occur adjacent to the synapse [15,16,17]. For example, the Fragile X Mental Retardation Protein (FMRP) is a powerful regulator of translation at the synapse [18]. At baseline, the phosphorylation of FMRP inhibits the translation of protein, and it is believed that a transient decrease in the phosphorylation of FMRP is responsible for the translational events that result in synaptic plasticity [19,20]. FMRP is also an endpoint effector of the mGluR5 signaling cascade [21]. Our lab has shown that 5 min following an aggressive experience there is a transient decrease in the phosphorylation of FMRP in the nucleus accumbens in female hamsters, followed by an increase (i.e., re-phosphorylation) 15 min following aggressive interactions [10]. However, nothing is known about the intracellular signaling events that regulate the aggression-induced phosphorylation state of FMRP in the nucleus accumbens. Furthermore, the intracellular signaling events in brain regions that regulate the nucleus accumbens, such as the medial prefrontal cortex, are also unexplored. Thus, the objective of the following study was to investigate the effects of aggressive experience on the phosphorylation of ribosomal S6 kinase (S6K), extracellular-signal related kinase 1/2 (ERK1/2), mammalian target of rapamycin (mTOR) and the expression of catalytic subunit of protein phosphatase 2A (PP2Ac) in the nucleus accumbens and the medial prefrontal cortex. To investigate this critical gap, we had female Syrian hamsters engage in up to five aggressive interactions with a male conspecific and assessed the expression of the phosphorylation of specific proteins in the forebrain following their last aggressive interaction (Figure 1A).

## 2. Results

### 2.1. Behavioral Plasticity for Two versus Five Consecutive Days of Aggressive Behavior

For female Syrian hamsters that had 2 consecutive days of aggressive behavior testing, there was no change in the latency to initiate an attack in the first compared to the second aggressive behavior test session (Figure 1B). However, female Syrian hamsters that experienced 5 consecutive days of aggressive behavior testing displayed a steady decrease in the latency to initiate an attack from the first to the fifth aggressive behavior test session (*F* = 5.876, *p* = 0.020, *df* = 1, 40) (Figure 1C). Specifically, the latency to the first attack was shorter on the fifth day of aggressive interactions compared to the first testing day (*d* = 1.477, *p* = 0.016, *df* = 8). See Supplementary Figure S1 for information on the total number of attacks. Ten minutes following the second or fifth day of aggressive behavior testing brain tissue was collected for protein analysis. 

#### 2.1.1. Effect of Aggressive Experience on the Phosphorylation and Expression of Kinases and Phosphatases of FMRP in the Nucleus Accumbens

**Figure 2:**
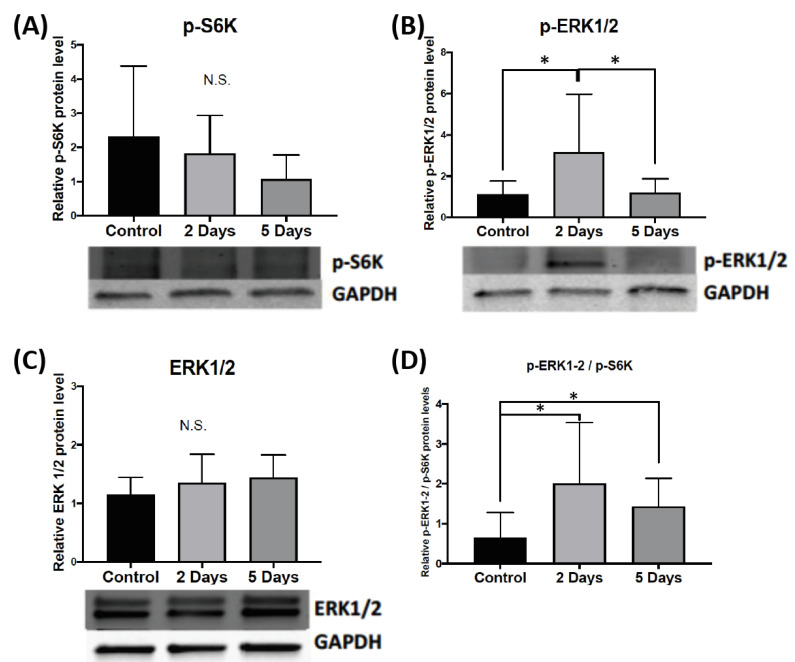
The effect of aggressive interactions on the phosphorylation and the expression of intracellular signaling molecules that regulate Fragile X Mental Retardation Protein (FMRP) in the nucleus accumbens (NAc). (**A**) There is no change in the phosphorylation of ribosomal S6 kinase (S6K) (phosphorylates FMRP) in the NAc 10 min following either 2 or 5 consecutive days of aggressive interactions compared to control female Syrian hamsters that had no social interaction experience (N.S.). (**B**) There is an increase in the phosphorylation of extracellular-signal related kinase 1/2 (ERK1/2) 10 min following 2 days of aggressive interactions compared to control female Syrian hamsters that had no social interaction experience and compared to levels following 5 days of aggressive interactions (* *p* < 0.05). (**C**) However, there was no change in the overall protein expression of ERK1/2 in the NAc (N.S.). (**D**) There is an increase in the ratio of the phosphorylation of ERK1/2 to the phosphorylation of S6K in the NAc following two and five days of aggressive interactions compared to female hamsters that had no social interaction experience (* *p* < 0.05). (Control n = 7–8, 2 Days n = 11, 5 Days n = 11). Data are presented as mean +/− standard deviation.

To determine if aggressive experience results in changes in the phosphorylation and expression of regulators of FMRP in the nucleus accumbens, we examined the phosphorylation of S6K (70 + 85 kDa), which in turn phosphorylates FMRP, and the expression of PP2Ac, which dephosphorylates FMRP. Ten minutes following either the second or the fifth consecutive day of aggressive experience there was no change in the phosphorylation of S6K in the nucleus accumbens compared to control females that had no social interaction experience (Figure 2A). There was also no change in the total protein expression of PP2Ac 10 min following either 2 or 5 days of aggressive experience compared to controls (Supplementary Figure S2).

#### 2.1.2. Effect of Aggressive Experience on the Phosphorylation and Expression of Upstream Signaling Molecules that Regulate FMRP Kinases and Phosphatases in the Nucleus Accumbens

To examine if aggressive experience results in changes in the phosphorylation of signaling molecules that regulate the activity of S6K and PP2Ac, we examined the phosphorylation of extracellular-signal related kinase 1/2 (ERK1/2) (42 + 44 kDa) and mammalian target of rapamycin (mTOR). Ten minutes following 2 consecutive days of aggressive experience there is an increase in the phosphorylation of ERK1/2 in the nucleus accumbens (*H* = 8.607, *p* = 0.014, *df* = 2) (Figure 2B) compared to both control females that had no social interaction experience (*d* = 1.001, *p* = 0.013, *df* = 16), and females that had 5 consecutive days of aggressive experience (*d* = 0.956, *p* = 0.012, *df* = 20). However, there was no change in the overall protein expression of ERK1/2 in the nucleus accumbens 10 min following either 2 or 5 consecutive days of aggressive experience compared to controls (Figure 2C). Ten minutes following either 2 or 5 days of aggressive experience there was no change in the phosphorylation of mTOR or total mTOR levels (Supplementary Figure S2).

To examine the relative balance between phosphorylated ERK 1/2 and phosphorylated S6K as a regulator of FMRP, we measured the ratio of the phosphorylation of ERK1/2 to S6K expression in the nucleus accumbens. Ten minutes following the second and fifth days of aggressive experience there is an increase in the ratio of the phosphorylation of ERK1/2 to S6K in the nucleus accumbens (*H* = 6.377, *p* = 0.041, *df* = 2) compared to control females (*d* = 1.170, *p* = 0.016, *df* = 16 for two days of aggression; *d* = 1.162, *p* = 0.038, *df* = 16 for five days of aggression) (Figure 2D).

#### 2.1.3. Effect of Aggressive Experience on the Phosphorylation and Expression of Secondary Signaling Molecules that Regulate FMRP Kinases and Phosphatases in the Medial Prefrontal Cortex

**Figure 3:**
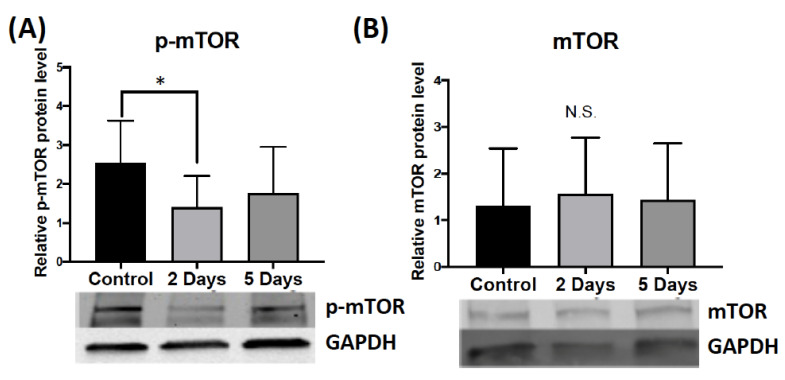
The effect of aggressive interactions on the phosphorylation and the expression of intracellular signaling molecules that regulate FMRP in the medial prefrontal cortex (mPFC). (**A**) There was a decrease in the phosphorylation of mammalian target of rapamycin (mTOR) (phosphorylates S6K) in the mPFC 10 min following 2 consecutive days of aggressive interactions compared to female Syrian hamsters that had no social interaction experience (* *p* < 0.05). (**B**) There was no change in the overall protein expression of mTOR in the mPFC 10 min following 2 consecutive days of aggressive interactions compared to female Syrian hamsters that had no social interaction experience (N.S.). (Control n = 7–8, 2 Days n = 9–11, 5 Days n = 8–11). Data are presented as mean +/− standard deviation.

Two or 5 consecutive days of aggressive experience did not affect the phosphorylation of ERK1/2 in the medial prefrontal cortex compared to control females that had no social interaction experience (Supplementary Figure S3). The ANOVA for phosphorylation of mTOR in the medial prefrontal cortex following aggressive experience did not show significant effects compared to control females, though the attendant post hoc test found a reduction after 2 days of testing (*d* = 1.178, *p* = 0.026, *df* = 17) (Figure 3A). There was no change in the overall protein expression of mTOR in the medial prefrontal cortex (Figure 3B). Finally, aggressive experience had no effect on the phosphorylation of S6K and no effect on the expression of PP2Ac in the medial prefrontal cortex (Supplementary Figure S3). 

### 2.2. Effect of Aggressive Experience on the Phosphorylation and Expression of the Translational Regulator FMRP in the Forebrain

Western blot analysis revealed that 10 min following either 2 or 5 consecutive days of aggressive experience there was no change in the phosphorylation of FMRP in the nucleus accumbens or prefrontal cortex compared to control females that had no social interaction experience (Supplementary Figure S4). For effects of aggressive experience on the phosphorylation and expression of secondary signaling molecules that regulate FMRP kinases and phosphatases in the caudate putamen see Supplementary Figure S5. 

## 3. Discussion

### 3.1. Intracellular Mechanisms of Translation

The goal of this study was to advance our understanding of the intracellular signaling mechanisms that underlie the neurological and behavioral adaptations resulting from the rewarding effects of aggressive interactions. There was a progressive decrease in the latency to display aggression in female Syrian hamsters that experienced 5 aggressive interactions. There is an increase in the phosphorylation of ERK1/2 in the nucleus accumbens 10 min following the second aggressive interaction. There is also a decrease in the phosphorylation of mTOR in the medial prefrontal cortex 10 min following the second aggressive interaction.

**Figure 4:**
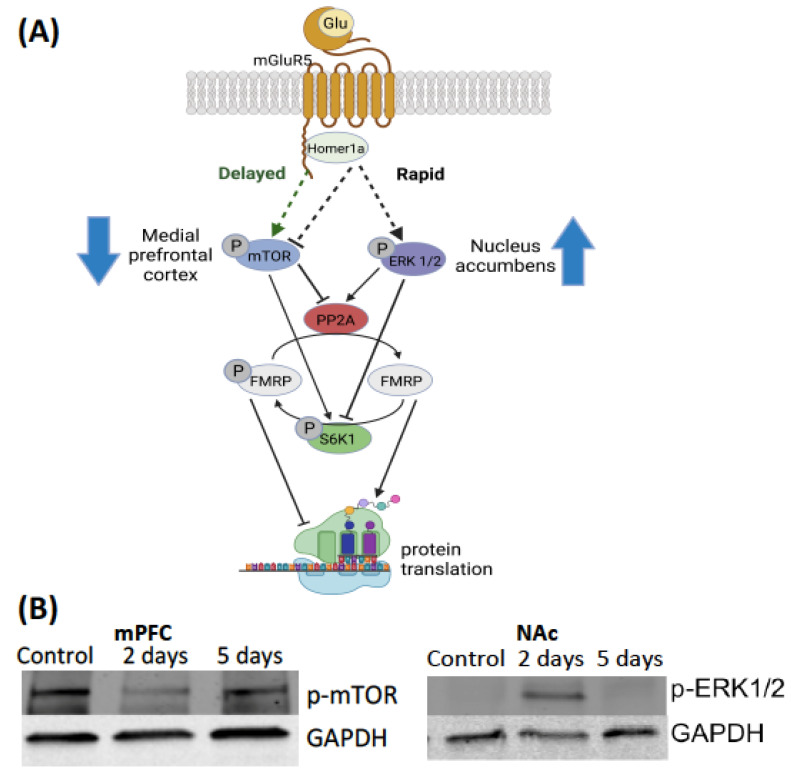
Hypothesized model of intracellular signaling molecules regulating FMRP phosphorylation and de-phosphorylation. (**A**) Shortly after aggressive interactions there is a decrease in the phosphorylation of mTOR in the mPFC and an increase in the phosphorylation of ERK1/2 in the NAc. Immediately following the activation of mGluR5 there is an increase in the activity of ERK1/2 and PP2Ac and a decrease in the activity of S6K in the nucleus accumbens. This pushes the balance of the activity of FMRP towards a decrease in phosphorylation, resulting in the translation of protein. However, in the medial prefrontal cortex following the activation of mGluR5 there is a decrease in the activity of mTOR, which would increase the activity of PP2Ac and decrease the activity of S6K. This also pushes the balance of the activity of FMRP towards a decrease in phosphorylation, resulting in the translation of protein. A delayed effect for both the nucleus accumbens and medial prefrontal cortex is an increase in the activity of mTOR, which then inhibits PP2Ac, but increases the activity of S6K. This pushes the balance of the activity of FMRP towards an increase in phosphorylation, resulting in the blocking of the translation of proteins. (**B**) Representative effect of the number of aggressive interactions on the phosphorylation of mTOR in the mPFC and ERK1/2 in the NAc 10 min following the last aggressive episode in female Syrian hamsters. From Figure 2 and Figure 3.

Figure 4 presents our model for intracellular signaling events following an aggressive encounter. In this model, the homeostatic balance of phosphorylation events regulates the activity of FMRP; kinases phosphorylate and thus activate FMRP, while phosphatases dephosphorylate and thus deactivate FMRP. In cell cultures it has been shown that the decrease in the phosphorylation of FMRP is driven by a rapid transient surge in the activity of PP2Ac [21,22]. The activation of mGluR rapidly elevates the enzymatic activity of PP2Ac and the decrease in the phosphorylation of FMRP [21]. However, little is known about potential signaling molecules that could regulate the increase in the activity of PP2Ac and the decrease in the phosphorylation of FMRP [19]. A potential candidate for driving the transient decrease in the phosphorylation of FMRP and resulting synaptic plasticity is the phosphorylation of ERK1/2 [23,24]. ERK1/2 regulates a wide variety of stimulated cellular processes, including proliferation, differentiation and survival [25,26,27].

Along a parallel pathway, the closure of this translational event is driven by a delayed and strong increase in the phosphorylation of the kinase S6K, which drives the re-phosphorylation (i.e., hyper-phosphorylation) of FMRP [24]. Furthermore, this increase in the phosphorylation of S6K is driven by the increase in the phosphorylation of mTOR [24]. This hyper-phosphorylation of FMRP thus inhibits the translation of proteins [24]. The sustained phosphorylation of ERK1/2 is also required for the hyper-phosphorylation of FMRP [24]. The phosphorylation of S6K is negatively correlated with the enzymatic activity of PP2Ac [24]. Additionally, the phosphorylation of mTOR also drives a decrease in the expression of PP2Ac [24] (Figure 4).

We present evidence that there is an increase in the phosphorylation of ERK1/2 in the nucleus accumbens 10 min following aggressive interactions. Interestingly, this effect was observed following the second aggressive interaction, but returned to baseline for hamsters that experienced five aggressive interactions. There was no change in the phosphorylation of mTOR, S6K, FMRP, or the enzymatic activity of PP2Ac. Although it did not reach significance, a trend for a decrease in the phosphorylation of S6K in the nucleus accumbens was noted. There was a significant increase in the ratio of the phosphorylation of ERK1/2 to the phosphorylation of S6K for hamsters that experienced two and five aggressive interactions compared to no aggressive interaction controls. ERK1/2 is a promising candidate for neural plasticity underlying aggression escalation due to its very well studied role in numerous fundamental cell functions [28,29,30]. For example, atrophy, or the decrease in the growth activity in muscle cells that comes with aging is associated with a decrease in contractile stimulated phosphorylation of ERK1/2. Interestingly, the contractile stimulated phosphorylation of mTOR and S6K in aged rats remains similar across the lifespan [25]. A recent study in male rats reported that 20 min following a conditioned place preference test that proceeded either social interaction conditioning or cocaine treatment did not result in a change in the phosphorylation of ERK1/2 in the nucleus accumbens [31]. However, a different study reported that 10 min following an acute social defeat experience in male mice there is an increase in the phosphorylation of ERK1/2 in the nucleus accumbens [32]. The increase in the phosphorylation of ERK1/2 was not observed 24 h following an acute social defeat. Thus, the phosphorylation of ERK1/2 in the nucleus accumbens is transient and potentially social behavior dependent. 

We also observed a decrease in the phosphorylation of mTOR in the medial prefrontal cortex 10 min following the second aggressive interaction. Similar to the increase in the phosphorylation of ERK1/2 in the nucleus accumbens, this effect was not observed for hamsters that experienced 5 aggressive interactions. There was no change in the phosphorylation of ERK1/2, S6K, FMRP, or the enzymatic activity of PP2Ac in the medial prefrontal cortex. Although it did not reach significance, a trend for an increase in the phosphorylation of ERK1/2 in the medial prefrontal cortex was noted. The phosphorylation of mTOR positively regulates the phosphorylation of the FMRP, the FMRP kinase S6K, and negatively regulates the activity of the FMRP phosphatase PP2Ac [33,34,35]. Thus, a transient decrease in the phosphorylation of mTOR may be a novel, non-canonical mechanism by which to induce translational events that induce long-lasting synaptic changes. mTOR signaling positively regulates mGluR mediated long-term synaptic activity and mushroom-shaped dendritic spines in the hippocampus in a Down’s syndrome rodent model [36]. To the best of our knowledge, this is one of the first published instances of changes in the molecular activity of intracellular regulators for social behavior. Interestingly, a recent study in male mice found that treatment of lysergic acid diethylamide (i.e., LSD) promotes social behavior via an increase in the phosphorylation of mTOR in the medial prefrontal cortex [37]. mTOR signaling also regulates social status in male African cichlid fish [38]. Collectively, these findings support transient intracellular signaling events in both the nucleus accumbens and the medial prefrontal cortex, but not in the caudate putamen, immediately following aggressive experience in female Syrian hamsters. Furthermore, the intracellular signaling mechanisms are divergent between the nucleus accumbens and the medial prefrontal cortex for aggressive interactions in females; there is an increase in the phosphorylation of ERK1/2 in the NAc, and a decrease in the phosphorylation of mTOR in the medial prefrontal cortex (mPFC) (Figure 4). Interestingly, research suggests that the ERK1/2 and mTOR pathways can both cross-activate or inhibit one another [27]. 

### 3.2. Heterogeneity of Intracellular Signaling

Previous studies have also reported divergent effects of the ERK1/2 and mTOR pathways on protein synthesis. These effects are also brain region specific. In mice with the genetic knockout of the gene that codes for FMRP (*FMR1*), there is elevated phosphorylation of ERK1/2, but not change in the phosphorylation of mTOR is observed in the neocortex. While elevated phosphorylation of mTOR, but no change in the phosphorylation of ERK1/2 is observed in the hippocampus in *FMR1 KO* mice [39]. In a separate study, in *FMR1 KO* mice there are increases in protein synthesis in the hippocampus, and this increased protein synthesis is blocked with mGluR5 or ERK1/2 inhibitors. An mTOR inhibitor had no effect on protein synthesis. ERK1/2 inhibition also blocked the increase in the seizure susceptibility associated with *FMR1 KOs* [23]. In human stem cells, *FMR1 KO* is associated with an increase in protein synthesis and the phosphorylation of ERK1/2 [40]. Thus, the effect of FMRP knockout often has divergent and independent effects on the ERK1/2 and mTOR signaling pathways. Furthermore, FMRP dysfunction on the activity of intracellular signaling can be both brain region and study specific. It is important to note that within the mesolimbic reward pathway the nucleus accumbens receives both dopaminergic and glutamatergic input, while the medial prefrontal cortex receives only dopaminergic input. These differences may contribute to the divergent effects in intracellular signaling observed.

### 3.3. Challenges of Interpretation

Aggressive experience results in long-term changes in the synaptic properties of dendritic spines on medium spiny neurons in the nucleus accumbens, along with behavioral adaptations. This study set out to investigate the transient intracellular signaling events responsible for these long-term changes. These studies are inherently challenging due to several factors. Social behavior is complex; it involves multiple sensory components and the sequential expression of many distinct micro-behaviors. We typically do not find correlates between the primary measures of aggression (attack latency and number of attacks) and intracellular signaling events ([10] and this study). One possibility is that the cellular plasticity is a consequence of the aggressive interactions (e.g., reward) and not driven by specific behavioral components of the aggressive interaction.

Another challenge relates to the timing of the signaling events. The changes in dendritic morphology occur multiple days following aggressive interactions and are long-term, but the intracellular signaling events are transient and occur within minutes. In this study we chose to investigate the changes in intracellular signaling 10 min following aggressive interactions to try to capture phosphorylation of as many proteins as possible. We already know that a decrease in the phosphorylation of FMRP following aggression can be observed at 5 min after aggression, with the re-phosphorylation of FMRP by 15 min after aggression [10]. Adding the results of this study to that previous work, it appears that the re-phosphorylation of FMRP is evident 10 min after aggression. What this suggests is that a more dynamic time-course is needed to sort out the pattern of phosphorylation of signaling proteins associated with aggression in our female hamster model.

## 4. Materials and Methods

### 4.1. Animals

Adult female (n = 30) and male Syrian hamsters (n = 16) (*Mesocricetus auratus*) were purchased from Charles River Laboratories (Wilmington, MA, USA) at approximately 60 days of age (120–130 g). Subject females were housed individually while intruder males were pair-housed in polycarbonate cages (females: 50.8 cm × 40.6 cm × 20.3 cm; males: 43.2 cm × 22.9 cm × 20.3 cm). These semi-natural housing arrangements maximize aggression in female hamsters, while decreasing aggression in male intruders [41,42,43]. All animals were maintained on a reversed 14:10 light/dark photoperiod (lights off at 1300 h) and all behavioral testing occurred during the first three hours of the dark phase. The animal room was maintained at a controlled temperature of 22 °C and food and water were available ad libitum. Hamsters acclimated for two weeks before experiments. All animal procedures were carried out in accordance with the National Institutes of Health Guide for the Care and Use of Laboratory Animals (NIH Publications No. 80-23; revised 2011) and approved by the University of Minnesota Institutional Animal Care and Use Committee.

### 4.2. Ovariectomy

To maximize aggressive behavior in females, circulating estradiol levels were maintained at low levels via bilateral ovariectomy [44]. Under sterile conditions, surgery was conducted using sodium pentobarbital anesthesia (Nembutal, 8.5 mg/100 g body weight, i.p.). Analgesic (Meloxicam, 1 mg/kg, s.c., Fort Dodge Animal Health, Overland Park, KS, USA) was administered prior to surgery and for 3 days of post-operative pain management. 

### 4.3. Behavioral Testing and Scoring

To mitigate potential experimental confounds, females were handled daily for at least four days prior to the first aggressive interaction session. A different adult male stimulus hamster was placed into a female subject’s home cage for 5 min each day, at the same time each day, and for either a 2- (n = 11) or 5-day (n = 11) period. Hamsters were weighed before behavior testing and the average body weight was balanced between treatment conditions (mean ~145 g per hamster per group). A two-step process was used to verify scoring (live scoring followed by video scoring). Each session was scored for two main criteria: (1) the latency to the subject’s first attack and (2) the total number of attacks. Attacks were operationally defined as the subject biting, or clearly attempting to bite, the intruder male. Intruder males were used at most every other day and for each test individual females were paired with a different male to minimize the likelihood that submission by the male would influence the behavior of the females [45]. The control females (i.e., no social interaction) did undergo the same handling and transport experiences as the experimental groups (n = 8). Two hamsters were removed from the study, as they did not display aggressive behaviors towards male intruders. 

### 4.4. Tissue Collection

Ten minutes following the second or fifth aggression test, females were rapidly decapitated without anesthetic. Control hamsters that did not experience social interaction also had tissue collected at this time. Coronal sections (approximately 1–2 mm) containing the nucleus accumbens (NAc), caudate putamen (CPu) and medial prefrontal cortex (mPFC) were taken with the aid of a brain matrix, and bilateral tissue punches (1 mm diameter) were immediately collected from each area (Supplementary Figure S6), referencing the hamster brain atlas [46]. NAc punches included the anterior commissure to bias the punches toward the NAc core, where we have previously found increases in dendritic spine density and glutamate receptors following aggressive experience [8,10]. Dorsal medial CPu punches were taken to evaluate the regional specificity of the biochemical changes within the striatum following aggressive experience. Punches were flash-frozen and stored in an −80 °C freezer.

### 4.5. Tissue Preparation

To prepare tissue for Western blot analysis, flash frozen tissue was first transferred into 1.5 mL protein lysate tubes with metallic beads. 100 μL of ice-cold RIPA buffer (250 mM NaCl and 50 mM Tris pH 8) and 1 μL of HALT phosphatase and 1 μL of HALT protease inhibitors (Thermo Fisher, Waltham, MA, USA) were added to each lysate tube. Tissue samples were then homogenized in a bullet blender (Next Advance, Inc., Troy, NY, USA) for 6 min. Twenty-one μL of detergent master mix (per 16 mL: 1 mL 100% triton, 10 mL of 10% sodium dodecyl sulfate (SDS) and 5 mL of 10% sodium deoxycholate) was then added to tubes with homogenized tissue. Samples were then rotated for 45 min with a sample mixer at 4 °C. Samples were centrifuged at 4^o^C for 20 min (12,000 rpm). Finally, the supernatant was aspirated (~80–100 μL) and placed in a fresh tube. Approximately 6 μL per sample was used for protein analysis and the rest was stored in a −80 °C freezer until used for Western blots. Protein was quantified using Bio-Rad protein DC assay (Bio-Rad Laboratories, Berkeley, CA, USA). Samples were run in triplicate at 20, 10 and 6.7 dilution factors (1.0–3.0 μL of sample and 19.0–17.0 μL of 1% SDS). Standards were run in duplicate in eight serial dilutions of 1.5–0.05 mg/mL (Bio-Rad Laboratories). 

### 4.6. Western Blot

For each subject 30 μg of total protein were added to a micro-centrifuge tube with 7.5 μL of 4× Laemmeli buffer (Bio-Rad Laboratories, Richmond, CA, USA) and RIPA buffer for a total volume of 30 μL and heated for 10 min on a 90 °C hot plate. Samples were then loaded into a 12–15% polyacrylamide gradient gel in 1% SDS running buffer (Mini-PROTEAN TGX Precast Mini Gel, Bio-Rad Laboratories, 110 mV for ~90 min). Proteins from the gel were then transferred to a nitrocellulose membrane (0.45 μm pore size, Bio-Rad Laboratories, Cat. # 1620115, 100 mV for 55 utes). After transfer, membranes were then blocked in a 50:50 dilution of Immobilon Block—PO Phosphoprotein Blocker (Millipore Sigma, Temecula, CA, USA, WBAVDP001) and TBST for 1 h. Membranes were then incubated overnight in primary antibodies diluted in TBST as indicated in Table 1. The following day, the membranes were washed in TBST for six 3 min washes. Membranes were then incubated in a Li-Cor incubation box for 1 h in the appropriate secondary antibodies (goat anti-rabbit 680RD IgG and goat anti-mouse 800CW IgG, 1:20,000, Li-Cor Biosciences, Lincoln, NE, USA) and then repeat washed in TBST six more times for 3 min each. Finally, membranes were imaged and analyzed using the Odyssey imaging system (Li-Cor Biosciences), as previous described [9]. Within each lane/subject, protein of interest was normalized to Glyceraldehyde 3-phosphate dehydrogenase (GAPDH). Within each gel, these relative expression values were then normalized to a control lane consisting of a pooled sample to reveal a relative expression value. Multiple proteins were assessed on the same gel: p-mTOR, p-S6K and p-ERK1/2 (Supplementary Figure S7); p-FMRP (Supplementary Figure S8) and PP2Ac; mGluR5, FMRP and Homer1a; and mTOR, S6K and ERK1/2, and thus were normalized to the same GAPDH band. For representative blots see Supplemental Figures S9–S11. For a summary table of antibodies see Table 1.

Different lanes were treated with either 30 or 15 μg of total protein to confirm that the antibodies were within the dynamic range of intensity expression (Supplementary Figure S12a). To confirm antibodies are sensitive to phosphorylation, specific lanes were treated with 2 μL of Lambda protein phosphatase, P9614-20KU, 2.25 μL of 10× manganese chloride, M0564, 2.25 μL of 10× lambda-ppase buffer, L9288 (Sigma Aldrich, ST. Louis, MO, USA) and 3.2 μL of Phosphatase, Alkaline from bovine intestinal mucosa, P0114-10KU (Sigma Aldrich, ST. Louis, MO, USA) at 30 °C for 30 min prior to the addition of 7.5 μL of 4× Laemmeli and prior to being heated for 10 min at 90 °C (Supplementary Figures S12b,c and S13).

### 4.7. Data Analysis

Data were analyzed using GraphPad Prism 8.3.0 for Windows. Statistical outliers were removed using the ROUT method (maximum False Discovery Rate, Q = 1%). All data were examined to determine if the assumptions of parametric statistical tests were met: normality (Bartlett’s test) and equal variance (Brown-Forsythe test). If assumptions were not met, a Kruskal–Wallis (non-parametric) test was performed (along with Dunn’s post hoc tests). All data are presented as mean ± standard deviation (SD).

Behavioral analyses: Paired *t*-tests or repeated-measures (RM) ANOVAs for linear trend were used to detect significant differences in aggressive behaviors following repeat aggressive experience across testing days (days 1 through 2 or 1 through 5 for the two corresponding groups), *p* < 0.05 [47]. 

Western blot analyses: For protein analyses, significant differences between groups were detected using one-way ANOVAs with LSD post hoc tests, *p* < 0.05.

## 5. Conclusions

We present data demonstrating that 10 min following two consecutive days of aggressive experience there is an increase in the phosphorylation of ERK1/2 in the nucleus accumbens, and a decrease in the phosphorylation of mTOR in the medial prefrontal cortex in female Syrian hamsters. Interestingly, these phosphorylation events are not observed in hamsters following five consecutive days of aggressive experience. A decrease in the latency to attack is observed after five days of aggressive experience, but not in the hamsters that received only two days of aggressive experience. We hypothesize that activation of mGluR5 by glutamate binding results in the rapid phosphorylation of ERK1/2, which increases the enzymatic activity of PP2Ac and inhibits the phosphorylation of S6K [48]. Thus, inhibiting the phosphorylation of FMRP, the FMRP homeostatic balance is pushed towards a decrease in phosphorylation and thus an increase in the translation of proteins, allowing changes in synaptic structure in the nucleus accumbens (e.g., mushroom-like spines, PSD-95, mGluR5 and AMPARs). We also hypothesize that in the medial prefrontal cortex, the transient translation of proteins following aggressive experience in females occurs via a transient decrease in the phosphorylation of mTOR. A decrease in the phosphorylation of mTOR decreases the activity of S6K, S6K is responsible for the phosphorylation of the FMRP. 

Thus, the transient increase in the phosphorylation of ERK1/2 in the nucleus accumbens and the decrease in the phosphorylation of mTOR in the medial prefrontal cortex may underlie the translational changes in these reward brain regions that drive the long-term morphological (mushroom-like spines) and thus electrical (glutamate release, PSD-95, mGluR5 and AMPARs) adaptations underlying aggressive experience [9,10,11]. Finally, these synaptic changes mediate the rewarding consequences of aggressive interactions and thus the corresponding decrease in the latency to display aggression with aggressive experience, i.e., the behavioral adaptations of reward encoding, in future encounters. Aggression is clearly a normal component of social interactions across species, including people (e.g., assertiveness). It is the escalation of aggression to psychological abuse or physical harm (e.g., domestic violence) that needs to be controlled. Our research is designed to identify mechanisms underlying the escalation of aggression as measured by a decreased latency to aggression onset. Molecular targets that can delay the initiation of aggression are valuable components of a therapeutic strategy for escalated aggression. With careful consideration of dosage and contraindications, therapeutics targeting the phosphorylation state of ERK1/2 [49,50] and mTOR [51,52] may be potential clinical options for regulating aggression, its rewarding consequences and ultimately mental health. 

## Figures and Tables

**Table 1:** Table of primary antibodies, concentration, company and catalog number utilized for this study.

Primary Antibody	Concentration	Company	Cat. #
rabbit anti-phospho-p70 S6 Kinase (Thr421/Ser424)	1:667	Cell Signaling	9204S
rabbit anti-PP2A C Subunit	1:10,000	Cell Signaling	2038S
rabbit anti-phosphop44/42 –MAPK (Erk1/2) (Thr202/Tyr204) (D13.14.4E) XP	1:667	Cell Signaling	4370S
rabbit anti-phospho-mTOR (Ser2448)	1:1000	Cell Signaling	2971S
rabbit polyclonal to FMRP (phospho S499)	1:500	Abcam	ab183319
rabbit anti-FMRP (Ser500) Polyclonal	1:500	Bioss Antibodies	BS013188R
rabbit anti-p70 S6 Kinase	1:1000	Cell Signaling	9202S
rabbit anti-p44/42 MAPK (Erk1/2)	1:20,000	Cell Signaling	9102S
rabbit anti-mTOR	1:10,000	Cell Signaling	2983S
rabbit anti-FMRP	1:750	Cell Signaling	4317S
mouse anti-GAPDH	1:10,000	Novus Biologicals LLC	b8245
mouse anti-GAPDH	1:20,000	Abcam	ab8245

## Data Availability

Raw data are available from the corresponding author upon request.

## Supplementary Materials

The following supporting information can be downloaded at: https://www.mdpi.com/article/10.3390/ijms24021379/s1
