# Supplementary material for: Aggression Results in the Phosphorylation of ERK1/2 in the Nucleus Accumbens and the Dephosphorylation of mTOR in the Medial Prefrontal Cortex in Female Syrian Hamsters
Source: Int J Mol Sci. 2023 Jan 10;24(2):1379. doi: 10.3390/ijms24021379 (PMC9862940; doi:10.3390/ijms24021379)
Supplement: Supplementary file 1 [file ijms-24-01379-s001.zip › ijms-2010987-supplementary.pdf]

Supplementary Material

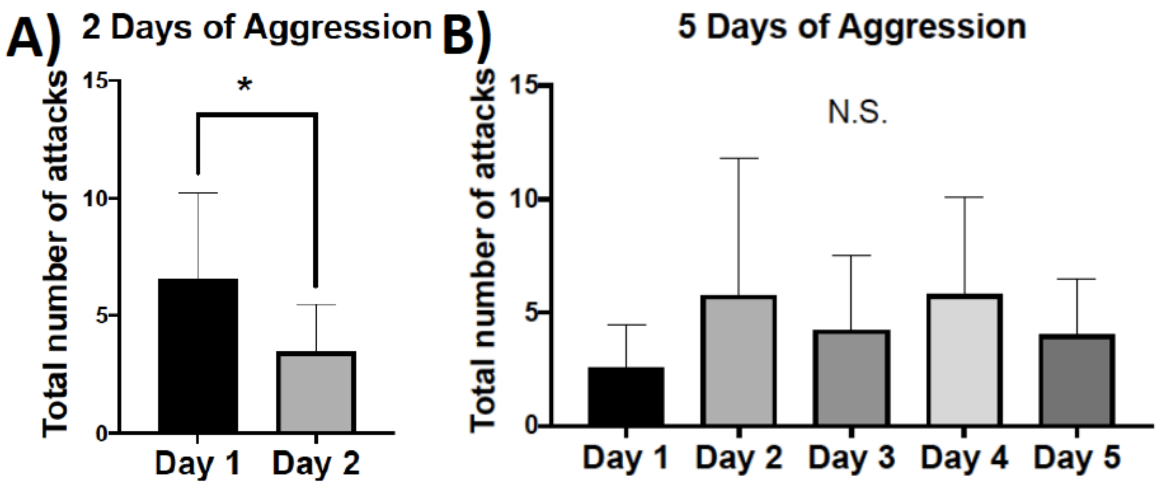

Figure S1: Total number of attacks over two and five consecutive days of aggressive interactions. **A)** For females that experienced 2 consecutive days of aggressive interactions, there was an unexpected decrease in the total number of attacks in the second compared to the first aggressive behavior test day (t=2.833, p=0.018, df=10). **B)** For females that experienced 5 consecutive days of aggressive experience, there were no differences in the total number of attacks over the five days of aggressive interactions (non-significant = N.S.). (* Indicates significant difference between groups, $p<0.05$). Data are presented as mean +/- standard deviation.

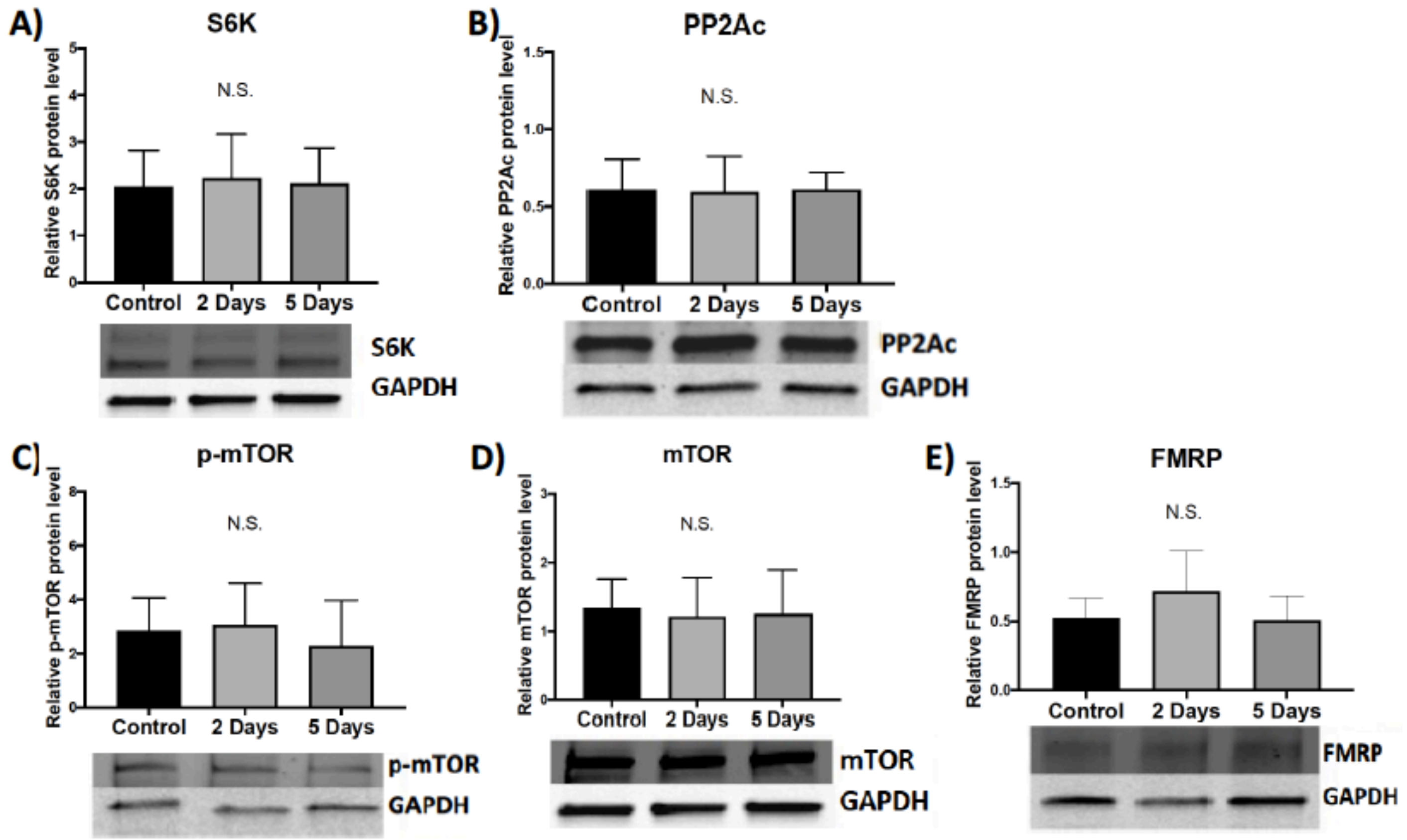

Figure S2: The effect of aggressive interactions on the phosphorylation and the overall protein expression of S6K, PP2Ac, mTOR and FMRP in the NAc. **A)** There was no change in the overall protein expression of S6K in the nucleus accumbens ten min following either two or five consecutive days of aggressive interactions compared to no social interaction controls (N.S.). **B)** There was also no change in the overall protein expression of PP2Ac (dephosphorylates FMRP) in the NAc (N.S.). **C)** There was no change in the phosphorylation of mTOR (phosphorylates S6K) in the NAc 10 min following either 2 or 5 consecutive days of aggressive interactions compared to control female Syrian hamsters that had no social interaction experience (N.S.). **D)** There was also no change in the overall protein expression of mTOR in the nucleus accumbens ten min following either two or five consecutive days of aggressive interactions compared to no social interaction controls (N.S.). **E)** There was also no change in the overall protein expression of FMRP in the nucleus accumbens (N.S.). (Control n = 7-8, 2 Days n = 10-11, 5 Days n = 10-11). Data are presented as mean +/- standard deviation. Representative Blots can be found in Supplementary Figure 9.

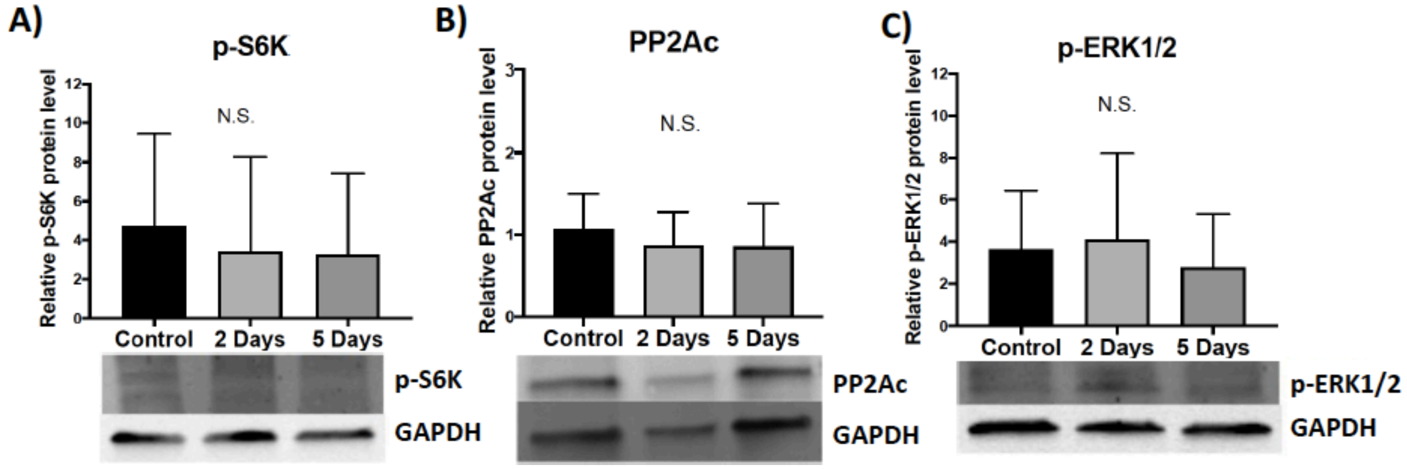

Figure S3: The effect of aggressive interactions on the phosphorylation and overall protein expression of S6K, PP2Ac and ERK1/2 in the PFC. **A)** There is no change in the phosphorylation of S6K (phosphorylates FMRP) in the mPFC 10 min following either 2 or 5 consecutive days of aggressive interactions compared to control female Syrian hamsters that had no social interaction experience (N.S.). **B)** There was also no change in the overall protein expression of PP2Ac (dephosphorylates FMRP) in the mPFC (N.S.). **C)** There was no change in the phosphorylation of ERK1/2 in the mPFC 10 min following aggressive interactions compared to control female Syrian hamsters that had no social interaction experience (N.S.). (Control n = 7-8, 2 Days n = 9-11, 5 Days n = 8-11). Data are presented as mean +/- standard deviation. Representative Blots can be found in Supplementary Figure 10.

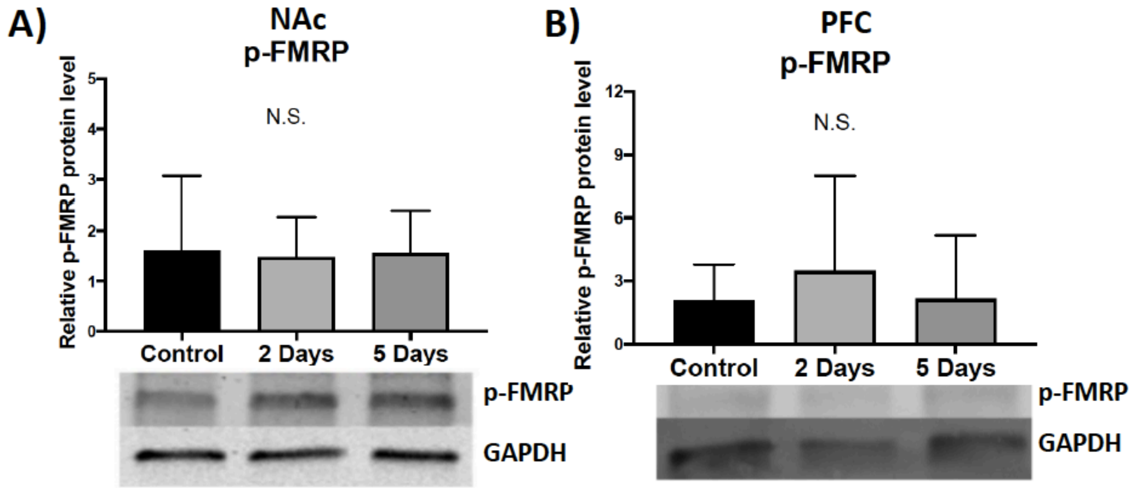

Figure S4: The effect of aggressive interactions on the phosphorylation of FMRP in the NAc and the mPFC. **A)** There is no change in the phosphorylation of FMRP in the NAc 10 min following either 2 or 5 consecutive days of aggressive interactions compared to control female Syrian hamsters that had no social interaction experience (N.S.). **B)** There is no change in the phosphorylation of FMRP in the mPFC 10 min following either 2 or 5 consecutive days of aggressive interactions compared to control female Syrian hamsters that had no social interaction experience (N.S.). (Control n = 7-8, 2 Days n = 11, 5 Days n = 11). Data are presented as mean +/- standard deviation.

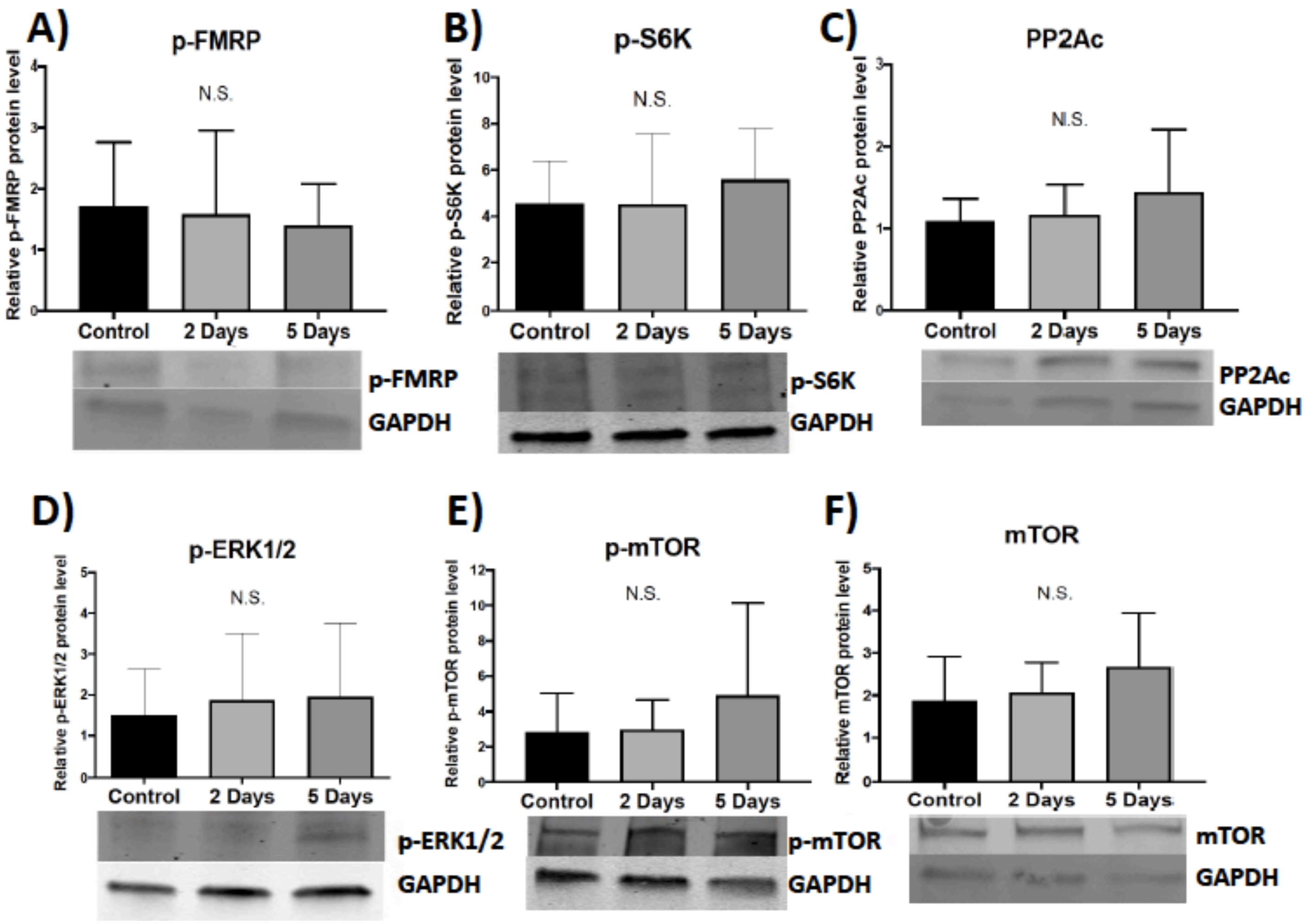

Figure S5: The effect of aggressive interactions on the phosphorylation and the expression of intracellular signaling molecules that regulate FMRP in the caudate putamen. **A)** Ten min following either 2 or 5 consecutive days of aggressive interactions there was no change in the phosphorylation of FMRP in the caudate putamen compared to control females that had no social interaction experience (N.S.). **B)** Ten min following either 2 or 5 consecutive days of aggressive interactions there was no change in the phosphorylation of S6K in the caudate putamen compared to control females that had no social interaction experience (N.S.). **C)** There was also no change in the overall protein expression of PP2Ac in the caudate putamen (N.S.). **D)** Ten min following either 2 or 5 consecutive days of aggressive interactions there was no change in the phosphorylation of ERK1/2 in the caudate putamen compared to control females that had no social interaction experience (N.S.). **E)** There was also no change in the phosphorylation of mTOR in the caudate putamen due to aggressive interactions (N.S.). **F)** There was also no change in the overall protein expression of mTOR in the caudate putamen across treatment conditions (N.S.). (Control n = 7-8, 2 Days n = 10-11, 5 Days n = 10-11). Data are presented as mean +/- standard deviation. Representative Blots can be found in Supplementary Figure 11.

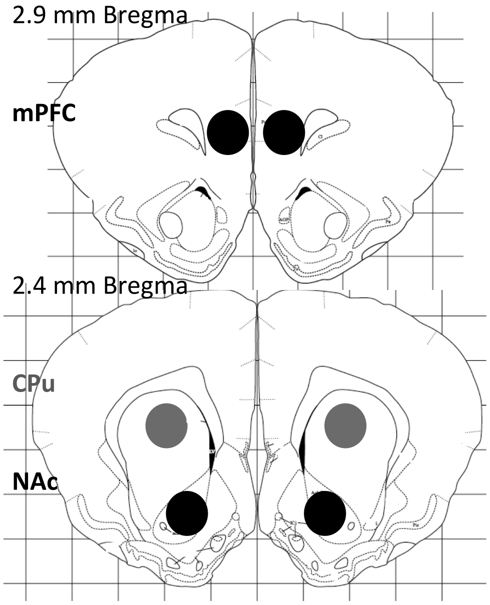

Figure S6: Tissue collection. Diagram of medial prefrontal cortex (mPFC), caudate putamen (CPu) and nucleus accumbens (NAc) bilateral tissue punches. Template of diagrams from Morin and Wood 2001.

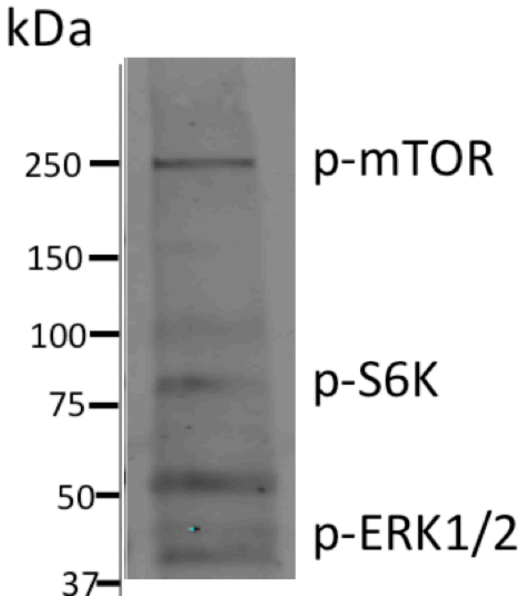

Figure S7: Representative lane for phosphorylated (p)-mTOR (signal detected at ~250kDa), p-S6K (signal detected at ~85 and 70kDa) and p-ERK1/2 (signal detected at ~44 and 42kDa).

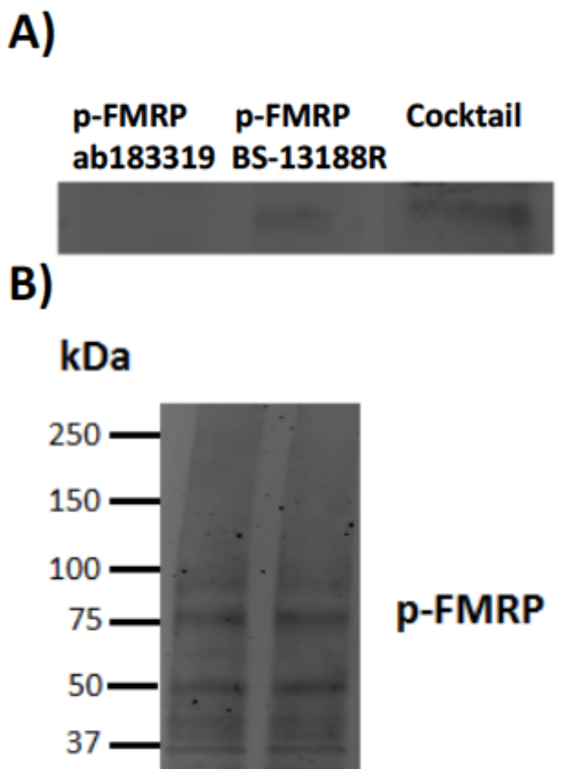

Figure S8: Combination of polyclonal anti-phosphorylated FMRP antibodies. **A)** First lane: 1:500 dilution of rabbit polyclonal to FMRP (phospho S499) (Abcam, Cambridge, MA, USA, ab183319). Second lane: 1:500 dilution of rabbit anti-FMRP (Ser500) Polyclonal (Bioss Antibodies, Woburn, MA, USA, BS013188R). Third lane: combination of 1:500 dilution of ab183319 and 1:500 dilution of BS013188R. **B)** Combination of 1:500 dilution of ab183319 and 1:500 dilution of BS013188R on a gel. Signal detected at ~75kDa.

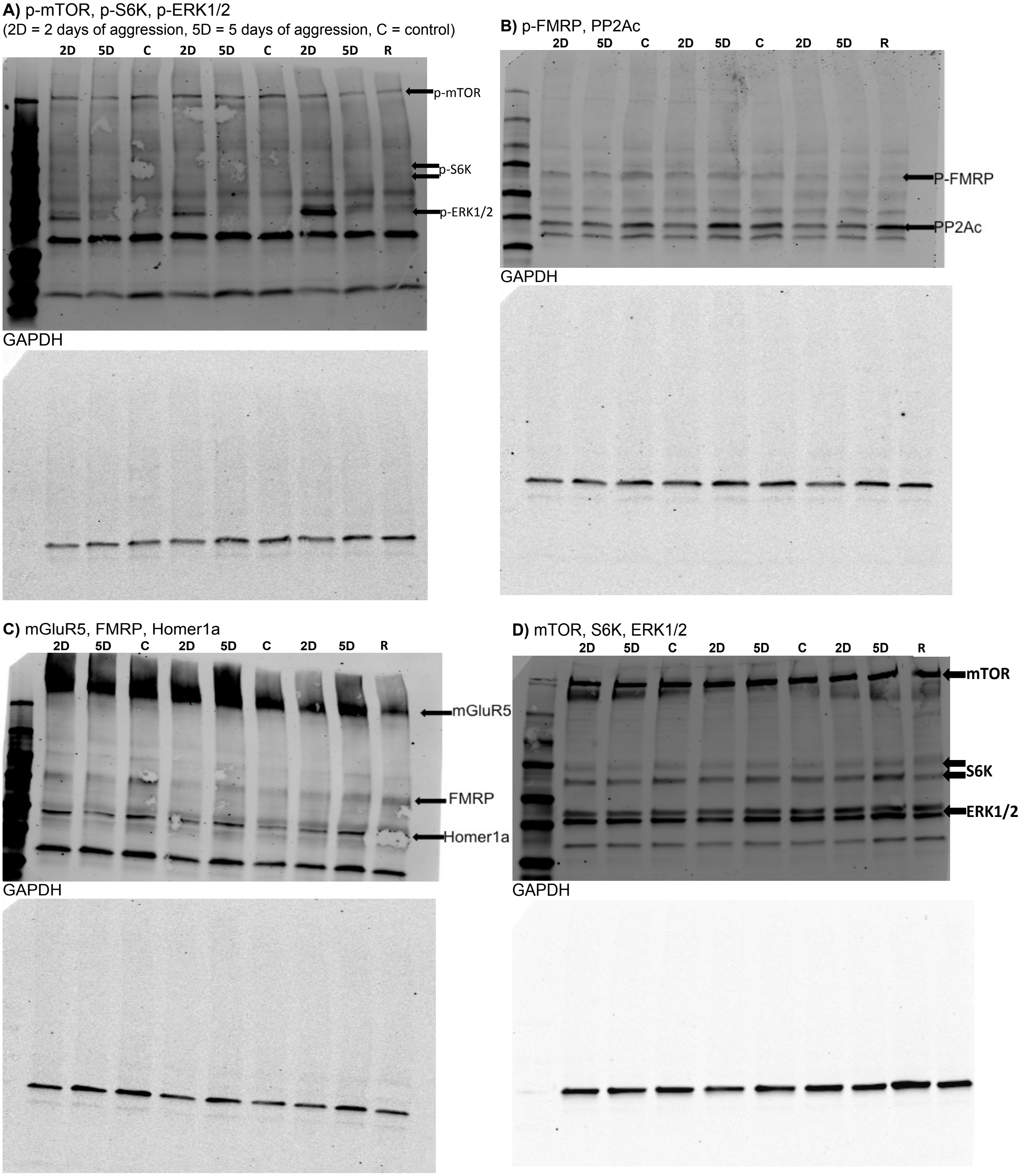

Figure S9: Original nucleus accumbens gels: **A)** p-mTOR, p-S6K, p-ERK1/2, GAPDH; **B)** p-FMRP, PP2Ac, GAPDH; **C)** mGluR5, FMRP, Homer1a, GAPDH; **D)** mTOR, S6K, ERK1/2, GAPDH. (2D = 2 days of aggression, 5D = 5 days of aggression, C = control, no aggressive interactions, R = reference, pooled sample).

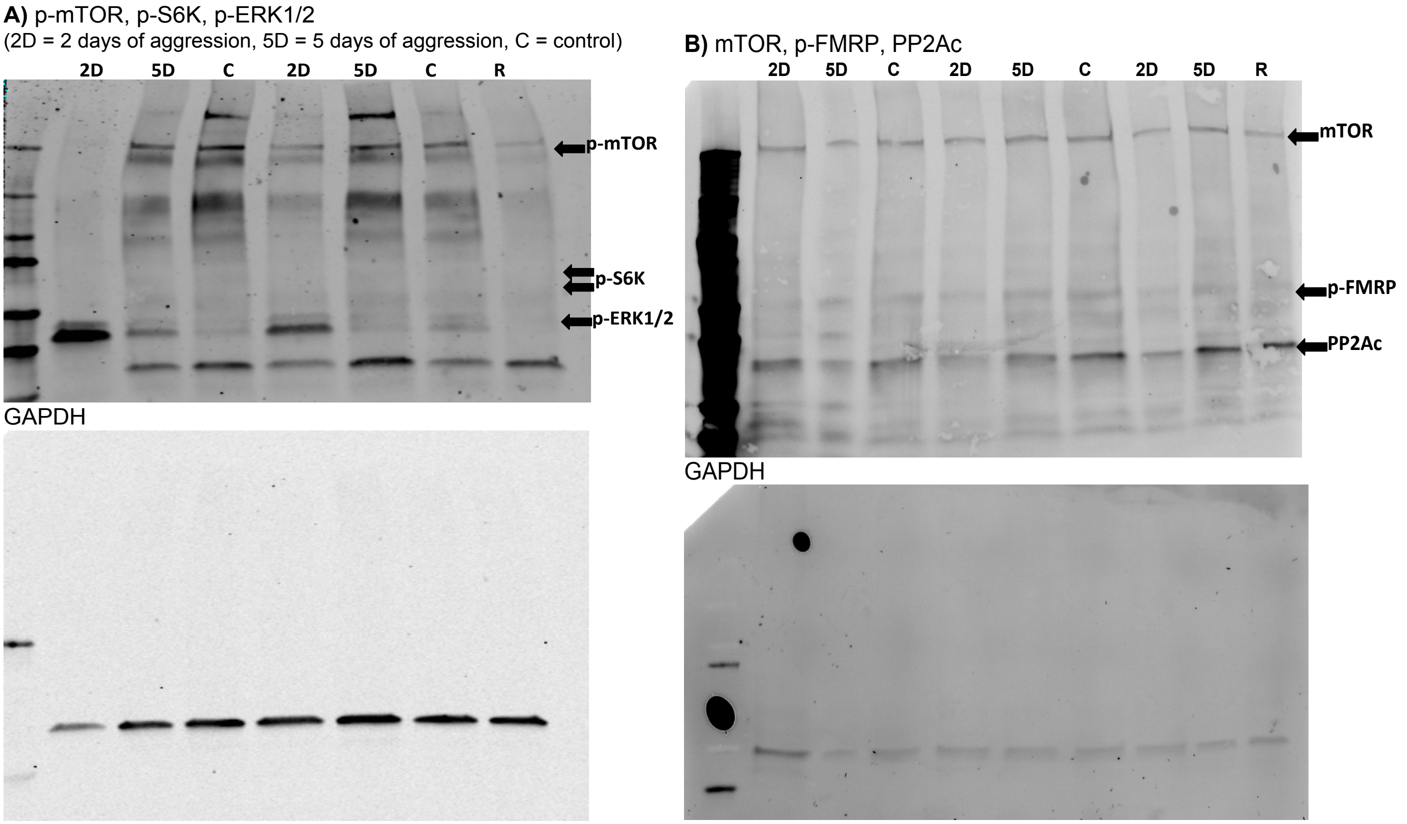

Figure S10: Original medial prefrontal cortex gels: **A)** p-mTOR, p-S6K, p-ERK1/2, GAPDH; **B)** mTOR, p-FMRP, PP2Ac, GAPDH. (2D = 2 days of aggression, 5D = 5 days of aggression, C = control, no aggressive interactions, R = reference, pooled sample).

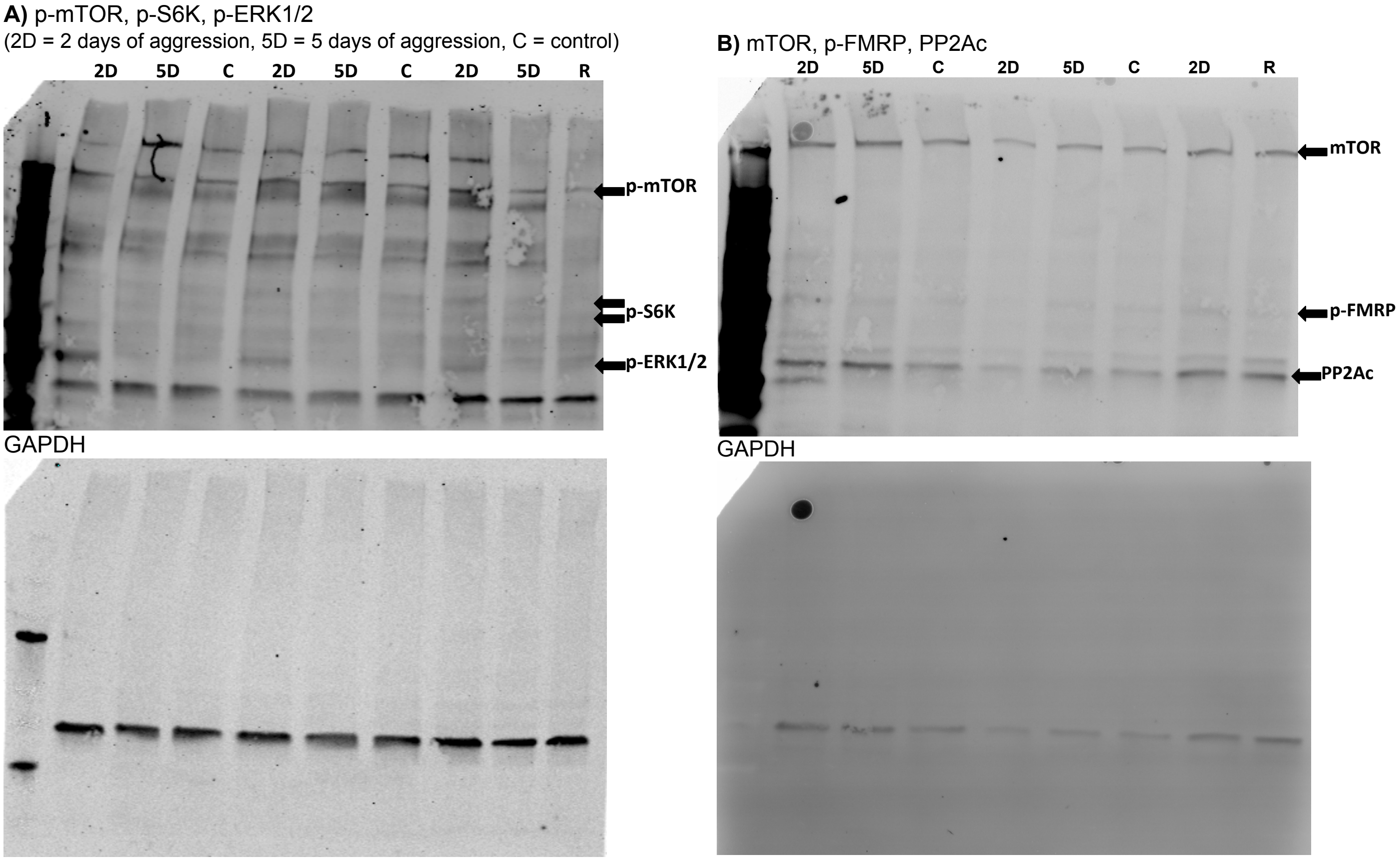

Figure S11: Original caudate putamen gels: **A)** p-mTOR, p-S6K, p-ERK1/2, GAPDH; **B)** mTOR, p-FMRP, PP2Ac, GAPDH. (2D = 2 days of aggression, 5D = 5 days of aggression, C = control, no aggressive interactions, R = reference, pooled sample).

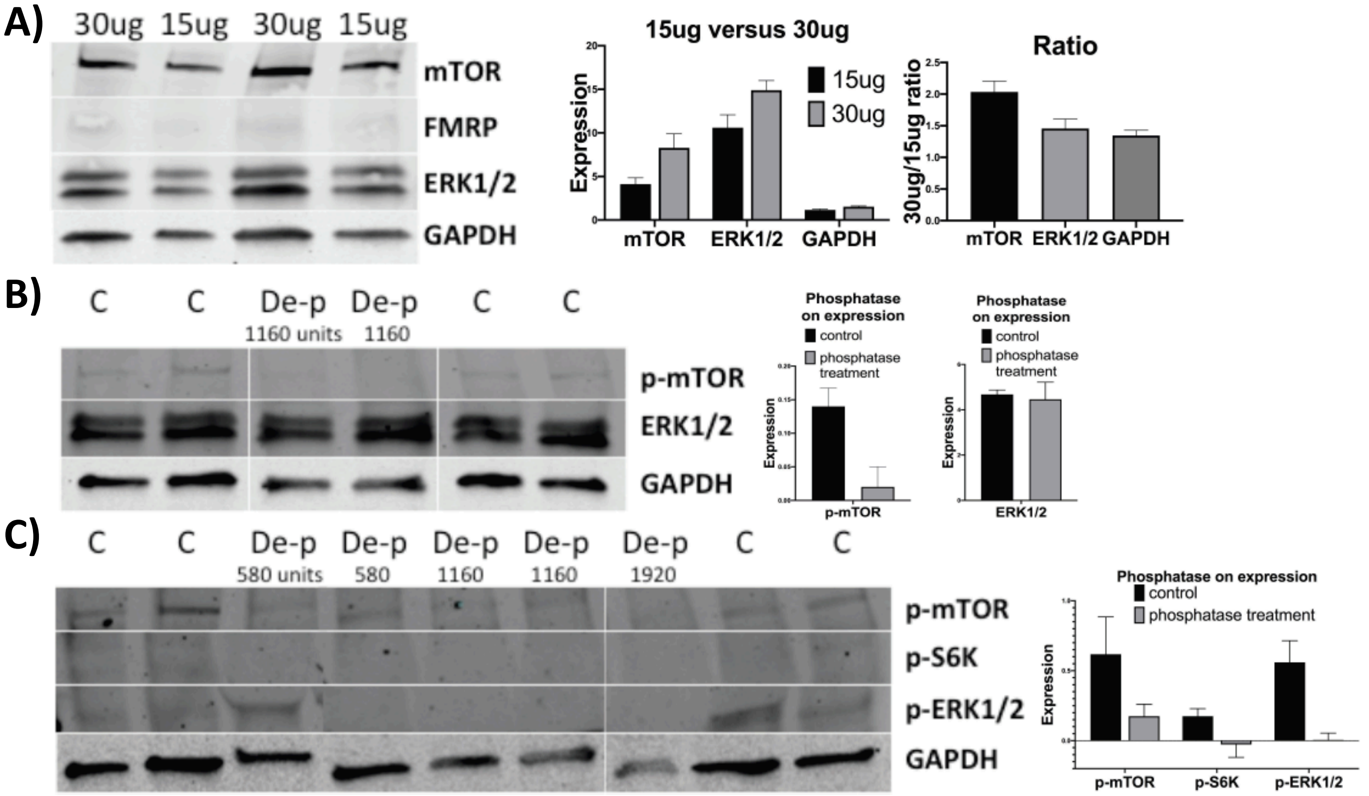

Figure S12: Validation of phoso-antibodies. **A)** Confirmation that antibody dilutions for mTOR, FMRP ERK1/2 and GAPDH are in the dynamic range of intensity sensitivity. Lanes 1 and 3 contained 30ug of protein; lanes 2 and 4 contained 15ug of protein. **B)** Confirmation of phosphorylation sensitivity of antibodies. Middle two lanes, i.e. lanes 3 and 4, are treated with dephosphorylate (De-p; amount of phosphorylates indicated in units). Gel was run against antibodies for p-mTOR and ERK1/2. Phosphorylate decreased the expression of p-mTOR, but not ERK1/2 or GAPDH. **C)** Lanes 3-7 were treated with dephosphorylate (De-p). Gel was run against antibodies for p-mTOR, p-S6K and p-ERK1/2. Phosphorylate decreased the expression of p-mTOR, p-S6K and p-ERK1/2, but not GAPDH. Data are presented as mean +/- standard error of the mean.

A) 15ug versus 30ug: mTOR, S6K, ERK1/2, GAPDH

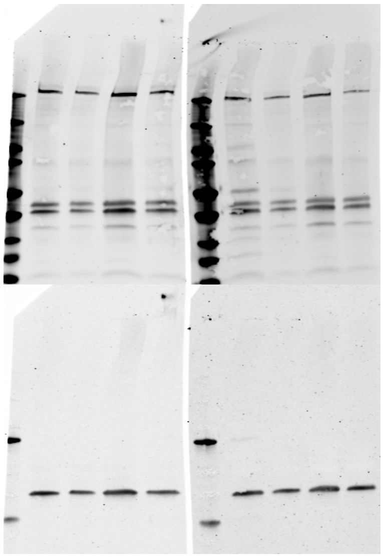

B) Dephosphorylate: p-mTOR, p-S6K, p-ERK1/2, GAPDH

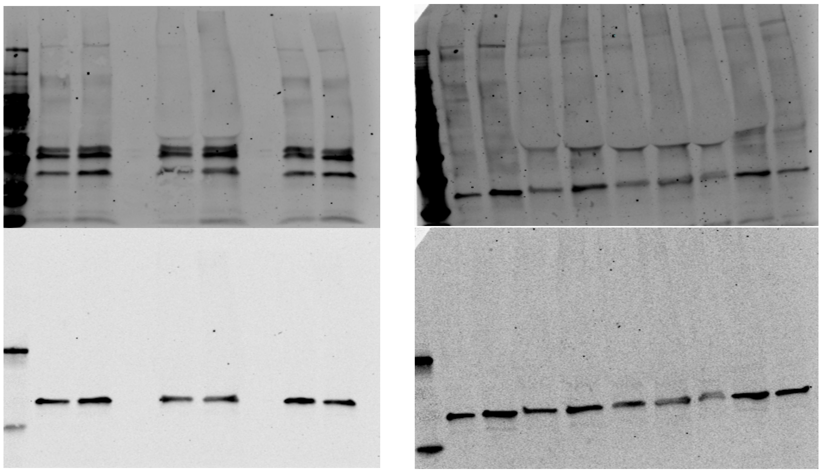

Figure S13: Original linear dynamic range and dephosphorylate gels: **A)** mTOR, FMRP, ERK1/2, GAPDH; **B)** p-mTOR, ERK1/2, GAPDH; p-mTOR, p-S6K, p-ERK1/2, GAPDH.
